# Supplementary material for: Using Polygenic Risk Scores Related to Complex Traits to Predict Production Performance in Cross-Breeding of Yeast
Source: J Fungi (Basel). 2022 Aug 29;8(9):914. doi: 10.3390/jof8090914 (PMC9500933; doi:10.3390/jof8090914)
Supplement: Supplementary file 1 [file jof-08-00914-s001.zip › jof-1840682-supplementary.pdf]

Table S1: Growth conditions tested for phenotyping in training set

**Index**	**Name**	**Media Composition**
1	YPACETATE	2% bactopeptone; 1% yeast extract; 2% acetate; 2% agar
2	YPDCAFEIN40	YPD; caffeine 40mM
3	YPDHU	YPD; hydroxyurea 30mg/ml
4	YPETHANOL	YPD; ethanol 15%
5	YPD14	14℃, 2% bactopeptone; 1% yeast extract; 2% glucose; 2% agar
6	YPDCAFEIN50	YPD; caffeine 50mM
7	YPDKCL2M	YPD; KCl 2M
8	YPGALACTOSE	2% bactopeptone; 1% yeast extract; 2% galactose; 2% agar
9	YPD40	40℃, 2% bactopeptone; 1% yeast extract; 2% glucose; 2% agar
10	YPDCHX05	YPD; cycloheximide 0.5µg/ml
11	YPDLICL250MM	YPD; LiCl 250mM
12	YPGLYCEROL	2% bactopeptone; 1% yeast extract; 2% glycerol; 2% agar
13	YPD42	42℃, 2% bactopeptone; 1% yeast extract; 2% glucose; 2% agar
14	YPDCHX1	YPD; cycloheximide 1µg/ml
15	YPDMV	YPD; methylviologen 20mM
16	YPRIBOSE	2% bactopeptone; 1% yeast extract; 2% ribose; 2% agar
17	YPD6AU	YPD; 6-azauracile 600µg/ml
18	YPDCUSO410MM	YPD; CuSO4 10mM
19	YPDNACL15M	YPD; NaCl 1.5M
20	YPSORBITOL	2% bactopeptone; 1% yeast extract; 2% sorbitol; 2% agar
21	YPDANISO10	YPD; anisomycin 10µg/ml
22	YPDDMSO	YPD; DMSO 6%
23	YPDNACL1M	YPD; NaCl 1M
24	YPXYLOSE	2% bactopeptone; 1% yeast extract; 2% xylose; 2% agar
25	YPDANISO20	YPD; anisomycin 20µg/ml
26	YPDETOH	2% bactopeptone; 1% yeast extract; 2% ethanol; 2% agar
27	YPDNYSTATIN	YPD; nystatin 10µg/ml
28	YPDANISO50	YPD; anisomycin 50µg/ml
29	YPDFLUCONAZOLE	YPD; fluconazole 20µg/ml
30	YPDSDS	YPD; SDS 0.2%
31	YPDBENOMYL200	YPD; benomyl 200µg/ml
32	YPDFORMAMIDE4	YPD; formamide 4%
33	YPDSODIUMMETAARSENITE	YPD; sodium metaarsenite 2.5mM
34	YPDBENOMYL500	YPD; benomyl 500µg/ml
35	YPDFORMAMIDE5	YPD; formamide 5%

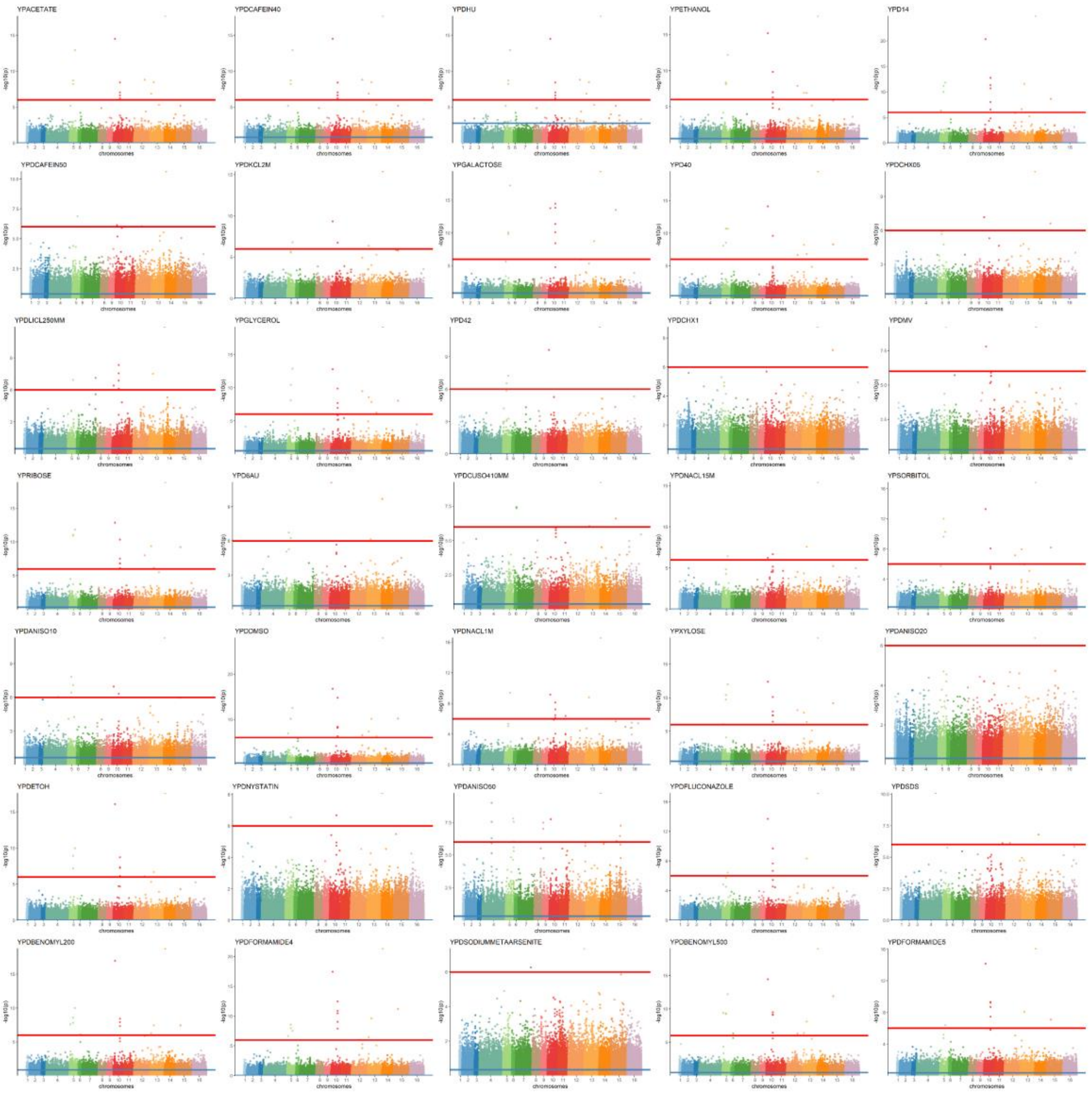

Figure S1: Genome Wide Association Study of 1011 isolates under 35 conditions. The red line indicates that p-value is $10e^{-8}$ and the threshold is statistically significant; the blue line indicates that p-value is 0.37885 and the threshold is the precise p-value threshold.

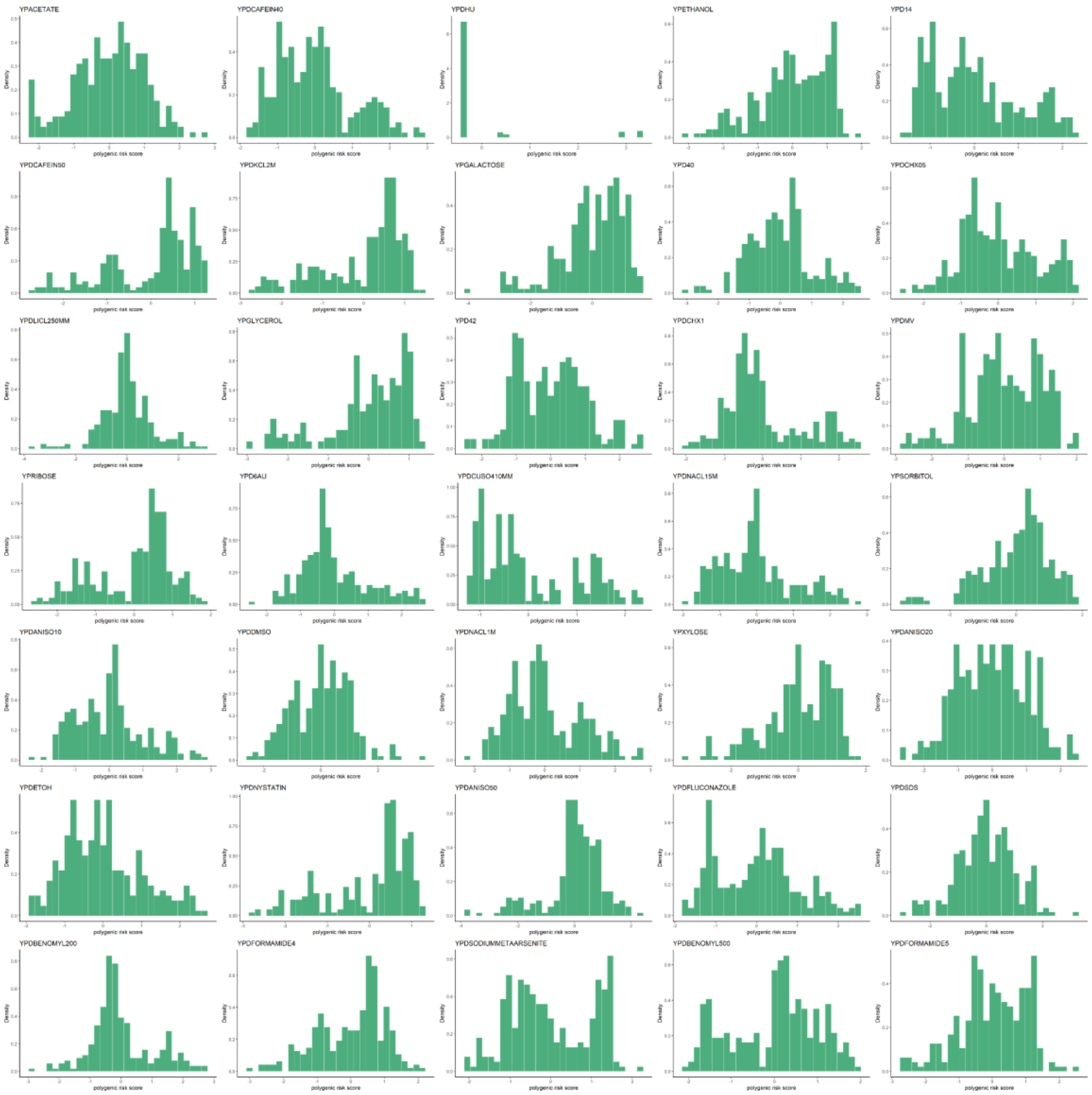

Figure S2. The distribution of polygenic risk score of 266 strains in testing set.
